# Vaccinia and Monkeypox Virus-Neutralizing Antibodies in People Living with HIV: A Serological Study in a Orthopoxvirus-Endemic, Low-Income Region in Brazil

**DOI:** 10.3390/pathogens14080733

**Published:** 2025-07-25

**Authors:** Thyago José Silva, Ana Gabriella Stoffella-Dutra, Victor Lacerda Gripp, Pollyana R. C. Gorgens, Iago José da Silva Domingos, Pedro Henrique Bastos e Silva, Bruna Caroline Chaves-Garcia, Erna Geessien Kroon, Etel Rocha-Vieira, Giliane de Souza Trindade, Danilo Bretas de Oliveira

**Affiliations:** 1 Laboratório de Doenças Infecciosas e Parasitárias, Universidade Federal dos Vales do Jequitinhonha e Mucuri, Diamantina 39100-000, Minas Gerais, Brazil; thyago.silva@ufvjm.edu.br (T.J.S.); victor.gripp@ufvjm.edu.br (V.L.G.); pollyanna.gorgens@ufvjm.edu.br (P.R.C.G.); etel.vieira@ufvjm.edu.br (E.R.-V.); danilo.bretas@ufvjm.edu.br (D.B.d.O.); 2 Laboratório de Vírus, Instituto de Ciências Biológicas, Departamento de Microbiologia, Universidade Federal de Minas Gerais, Belo Horizonte 31270-901, Minas Gerais, Brazil; anagstoffella@gmail.com (A.G.S.-D.); iago.jsd@gmail.com (I.J.d.S.D.); pedrohebastos@gmail.com (P.H.B.e.S.); ernagkroon@gmail.com (E.G.K.); gitrindade@yahoo.com.br (G.d.S.T.)

**Keywords:** orthopoxvirus, vaccination, cross protection, human immunodeficiency virus

## Abstract

Co-infections of Orthopoxviruses (OPVs), such as vaccinia virus (VACV) and monkeypox virus (MPXV), and the human immunodeficiency virus (HIV) can be associated with severe outcomes. Serro’s dairy region, located in Minas Gerais, southeastern Brazil, is an endemic area for VACV, where zoonotic outbreaks affect rural communities. This epidemiological context is especially relevant for at-risk populations, such as people living with HIV (PLHIV). This study aimed to assess the presence of neutralizing antibodies (NAbs) against OPV in PLHIV in this endemic setting. Serum samples were collected from 177 PLHIV in treatment at the specialized service between December 2021 and August 2022. VACV and MPXV NAbs were measured using the plaque reduction neutralization test (PRNT) and VACV-infected cells. The overall occurrence of OPV NAbs was 27.7%. NAbs were higher in individuals born before 1980 (53.3%) than those born after 1980 (1.1%). Among anti-VACV-seropositive individuals, 40.8% also had MPXV NAbs, suggesting cross-immunity. These findings indicate the circulation of VACV in PLHIV and highlight the increased susceptibility to OPV infections among individuals born after the cessation of smallpox vaccination. The results reinforce the importance of continued surveillance of OPV, especially in endemic regions and vulnerable populations.

## 1. Introduction

The genus Orthopoxvirus (OPV), belonging to the family Poxviridae, comprises a variety of viruses known to cause diseases in humans and animals. This family of enveloped viruses is characterized by its large genomic size and complex structure, with a capsid that contains double-stranded DNA [1].

Zoonotic OPV within the Poxviridae family are known to cause outbreaks worldwide, including Orthopoxvirus vaccinia (VACV), Orthopoxvirus cowpox (CPV), and Orthopoxvirus monkeypox (MPXV). The causative pathogen of smallpox, Orthopoxvirus variola (VARV), is the only OPV restricted to humans, and historically, the smallpox eradication was possible due to the cross-immunity observed among Orthopoxviruses [2,3,4]. However, the waning of global population immunity to OPV since the discontinuation of VACV smallpox vaccination campaigns in the 1970s is a growing concern, especially in light of the repeated emergence events of zoonotic OPV [4,5]. In Brazil, the discontinuation of smallpox vaccination occurred in 1978. Smallpox was declared eradicated by the World Health Organization (WHO) in 1980 [4].

MPXV, another member of the Poxviridae family and Orthopoxvirus genus, is the causative agent of a neglected zoonosis that remained endemic to the sub-Saharan African region for decades until 2022, when outbreaks were reported in non-endemic countries such as Australia, Belgium, Canada, France, Germany, Italy, Portugal, Spain, Sweden, the United Kingdom, and the United States [6]. In Brazil, on 31 May 2022, the first suspected case in the country was reported and later confirmed [7]. In August 2024, MPXV was declared a Public Health Emergency of International Concern by the WHO, a global emergency that began in 2022. Thus, the viral circulation and the occurrence of MPXV human cases in Brazil highlight the importance of epidemiological surveillance and investigation of cross-protection immunity of OPV in Brazilian populations.

In Brazil, VACV is the causative agent of an emerging viral zoonosis known as bovine vaccinia (BV). The BV mainly affects the udders of cattle and the hands of milkers, causing vesiculo-pustular lesions on the hands and arms that can spread through self-inoculation to other parts of the body, such as the face, eyes, and genitals. The Brazilian southeastern region, particularly the state of Minas Gerais, is considered the epicenter of VACV circulation in the country.

The Serro microregion, in the northern part of Minas Gerais, is an essential endemic focus of BV [8]. The region has a dairy production circuit, known as the dairy region of Serro, which is particularly known for its traditional artisanal cheese production. As Brazil has the world’s largest commercial cattle herd, the Serro dairy region is economically and culturally significant. Endemic BV outbreaks in Brazil can affect hundreds of rural properties and result in substantial financial losses due to decreased milk production, mastitis, and other secondary bacterial infections [8].

In chronically immunodeficient individuals, VACV can lead to the development of a more severe and lethal condition, called progressive vaccinia, characterized by deep and uncontrolled growth of lesions [9,10]. Thus, studying the circulation of VACV in the population carrying the human immunodeficiency virus (HIV) in regions where VACV is endemic, such as the dairy region of Serro, is relevant.

People living with HIV (PLHIV) in the region are followed up at the Specialized Assistance Service (SAS) of Diamantina. This outpatient facility is part of the Brazilian program to address the HIV/AIDS epidemic. The facility serves as a referral center for 28 municipalities, including those within the dairy region of Serro, and provides healthcare services to an estimated population of 330,000 inhabitants. PLHIV utilize this service for regular exams and clinical monitoring.

The circulation of a virus within a population and the study of its risk and protective profiles can be evaluated through the screening for neutralizing antibodies (NAbs) against the virus amongst individuals in a population [11]. Thus, the present study was conducted to determine the frequency of anti-OPV NAbs and to investigate the cross-protection of VACV vaccination and/or natural infection against MPXV in PLHIV.

## 2. Materials and Methods

This study involved 177 PLHIV of both sexes, aged between 21 and 82 years, from 24 different municipalities, assisted by the SSAS of Diamantina, Minas Gerais, Brazil. During their routine examinations, between 14 December 2021 and 30 August 2022, individuals underwent blood sampling with informed and voluntary consent. Sociodemographic data, such as city of origin, race, gender, age group, marital status, education, and occupation, and information on virological and immunological control were extracted from medical records and a data collection instrument.

The study was approved by the Research Ethics Committee of the Universidade Federal dos Vales do Jequitinhonha e Mucuri (protocol number CAAE: 36223420.8.0000.5108).

To investigate the presence of NAbs against OPV in serum samples, plaque reduction neutralization tests (PRNTs) were conducted as previously described [12] with some modifications [13]. The assay is the gold standard for the differential detection of OPV antibodies, has high specificity, analytical reliability, and has been extensively employed in soroepidemiological investigations of anti-OPV NAbs in different animal species [11,13,14].

Initially, sera were screened in duplicate at a dilution of 1:20. The dilution was prepared in Eagle’s Minimum Essential Medium (MEM) (GIBCO^®^, Whaltam, MA, USA) without the addition of fetal bovine serum (FBS) and mixed with an equal volume of MEM containing between 100 and 150 plaque-forming units (PFUs) of intracellular mature virus (IMV) of VACV strain Western Reserve or MPXV clade II, isolated during the 2022 outbreak. Viruses were provided by the Virus Laboratory of Universidade Federal de Minas Gerais (UFMG), cultured under BSL-3 (Biosafety Level 3). Before dilution, complement system proteins were denatured by incubating the serum at 56° for 30 min. As a control, the diluted patient serum sample was replaced with FBS. The serum and virus solutions were mixed and incubated for approximately 16 h at 37°, 5% CO_2_. Six-well plates containing African green monkey (*Cercopithecus aethiops*) kidney BSC-40 cells [American Type Culture Collection (ATCC) CRL-2761] monolayers at 80% confluence were inoculated with virus/serum mix solutions and incubated at 37° for one hour, in 5% CO_2_. After adsorption, MEM with 2% FBS was added to each well, and the plates were incubated for 48 h at 37° and 5% CO_2_. After 48 h, when cytopathic effects were observed, the plates were fixed with 10% formalin and stained with 1% crystal violet (SYNTH^®^, Diadema, Brazil). Controls with infected and uninfected cells were included in each plate. To maintain the viability of the virus control, FBS was added to this solution at the same concentration (2.5%). The cell control contained 2.0% FBS media only. The viral lysis plaques were visualized and counted. Samples were considered positive if they showed an average reduction of at least 50% compared to the viral control used in the assay. Samples positive for VACV underwent PRNT assays for MPXV neutralization. The MPXV PRNT was conducted in a BSL-3 laboratory, following biosafety protocols.

A descriptive analysis of the qualitative data was performed, and Pearson’s chi-square test was applied to compare participants positive and negative for NAbs, using the SPSS™ statistical software (version 22). For quantitative data, expressed as mean and standard deviation, normality was verified using the Shapiro–Wilk test. Due to the absence of normal distribution, the nonparametric Mann–Whitney (for comparisons between two groups) and Kruskal–Wallis (for three or more groups) tests were used, followed, when appropriate, by Dunn’s post hoc test, using the GraphPad Prism software (version 8.0, GraphPad Software, La Jolla, CA, USA). Correlations between quantitative variables were assessed using the Spearman test. The map was generated using the QGIS™ program. A *p*-value ≤ 0.05 was considered statistically significant.

## 3. Results

**Figure 1:**
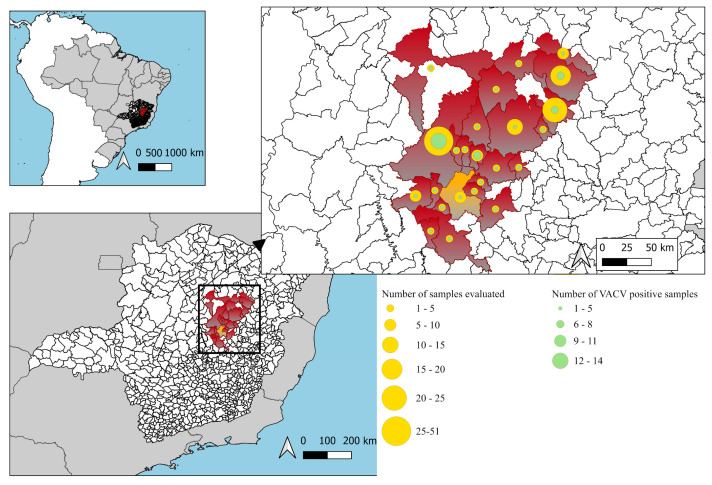
Number of individuals and VACV PRNT positive results by municipalities.

Of the 177 samples tested, 49 (27.7%) were positive, and 128 (72.3%) were negative for anti-VACV NAbs. Among the 49 positive samples, 20 (40.8%) also had anti-MPXV NAbs, while 29 (59.2%) were negative for MPXV NAbs. A descriptive analysis of the results and comparisons between participants with and without neutralizing antibodies was performed (Table 1). The percentages of viral neutralization were also compared between the different groups described in Table 1. Figure 1 shows the number of samples analyzed and the number of individuals with positive PRNT results for VACV by municipality in the BV-endemic region of northern Minas Gerais, Brazil.

**Figure 2:**
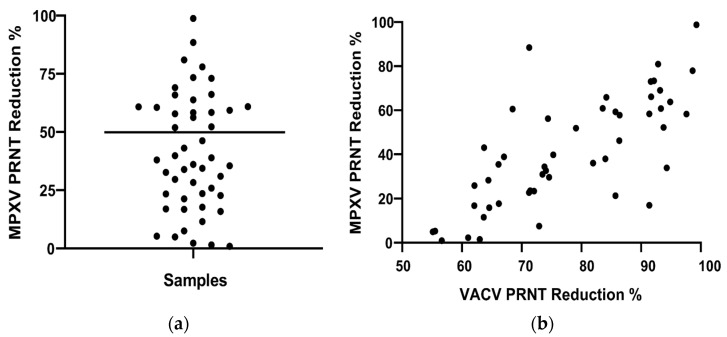
(**a**) Scatter plot of MPXV PRNT reduction percentage for serum samples of PLHIV (n = 49) related to MPXV virus control. Anti-MPXV NAbs were measured by 50% PRNT. The highlighted line represents the cutoff for positive and negative samples. (**b**) Comparison of PRNT reduction percentage results between MPXV and VACV PRNT (n = 49) related to the virus control.

The samples positive in the VACV PRNT assay were submitted to PRNT for MPXV. Of the 49 sera analyzed, 20 were positive, a frequency of 40.8% (Figure 2a). All the anti-MPXV NAb positive individuals were born before 1978, suggesting they may have been vaccinated against smallpox. No correlation was observed between percentage reduction for MPXV and age of the individuals (r = 0.27; *p* = 0.064), which may be due to age homogeneity of the group (mean = 57.3 ± 7.9 SD). Comparing the PRNT results between VACV and MPXV, a positive correlation was observed in the percentages of virus reduction (r = 0.73; *p* < 0.0001, Spearman correlation, Figure 2b). All samples showed a higher rate of reduction for VACV than for MPXV.

## 4. Discussion

The discontinuation of smallpox vaccination in Brazil in 1978 led to declining population immunity against OPV. Our study found that among individuals born before 1978, 53.3% tested positive for anti-OPV NAbs, with a higher percentage of PRNT reduction for VAVC than those born after that year. This positivity rate is consistent with studies conducted in Japan (50%) [15] and the USA (50%) [16]. These findings suggest that HIV does not impair the maintenance of memory humoral immunity against Orthopoxviruses in previously vaccinated individuals. However, among those born after smallpox eradication, only 1.1% had detectable NAbs, highlighting the lack of humoral protection against OPV in this population. An additional semiquantitative analysis could determine the anti-OPV antibody titers of the positive samples.

Our findings reinforce concerns about increased susceptibility to OPV infections following the cessation of vaccination, especially in immunocompromised groups such as PLHIV. Given the ongoing zoonotic emergence and re-emergence of OPV, including the recent global MPXV outbreak [4,5], the declining frequency of NAbs is particularly concerning. The presence of anti-OPV NAbs in PLHIV born after smallpox eradication suggests ongoing natural circulation of VACV, which could pose significant public health and economic burdens.

A key observation of this study was that none of the samples showed higher neutralization against MPXV than VACV. It indicates that all detected NAbs originated from exposure to VACV rather than MPXV. Previous reports showed that smallpox vaccination-induced NAbs exhibit cross-reactivity with MPXV [5,17]. Indeed, in our study, 40.8% of individuals with positive VACV PRNT results also neutralized MPXV, and all were born before 1978, further supporting the hypothesis that these antibodies originated from smallpox vaccination rather than natural MPXV exposure. Also, the individuals have no clinical or laboratory diagnoses of MPXV infection registered in their medical records. A Western blot analysis of viral lysate could determine antibodies specific to these different Orthopoxviruses.

PLHIV have been disproportionately affected by OPV outbreaks, particularly MPXV, with 38–50% of cases in the 2022 outbreak occurring in this group, often with severe clinical outcomes and higher hospitalization rates [18,19]. Hence, it is important to monitor this population in OPV-endemic regions, such as the northern region of Minas Gerais, Brazil. However, data on OPV cross-protection in HIV coinfections remain limited, and further investigation of VACV and MPXV circulation amongst this at-risk population is needed.

In conclusion, our serological screening in an HIV-positive cohort from an OPV-endemic area highlights the cross-protection of NAbs within the Orthopoxvirus group. The observed 27.7% frequency suggests that some HIV-positive individuals may retain partial protection against severe OPV infections. Future research should focus on longitudinal surveillance of anti-OPV NAbs in immunocompromised populations and identification of risk factors associated with OPV exposure. Sustained epidemiological monitoring is essential to support public health strategies for preventing and managing future OPV outbreaks.

## Figures and Tables

**Table 1:** Demographic and laboratory variables related to the seroprevalence of NAbs against OPV.

Variable	Total n (%)	Positive n (%)	Negative n (%)	*p*-Value	VACV PRNT % Media ± SD	*p*-Value
Gender						
Male	111 (62.7)	28 (25.2)	83 (74.8)	0.387 ^b^	33.6 ± 28.3	0.312 ^c^
Female	66 (37.3)	21 (31.8)	45 (68.2)		38.2 ± 30.6	
Birth year						
Before 1978	90 (50.8)	48 (53.3)	42 (46.7)	<0.0001 *^b^	51.7 ± 30.8	<0.0001 *^c^
After 1978	87 (49.2)	1 (1.1)	86 (98.9)		18.3 ± 13.5	
Self-Reported Skin Color						
White	26 (14.8)	10 (38.5)	16 (61.5)	0.359 ^a^	43.1 ± 28.3	0.1543 ^d^
Black	19 (10.8)	6 (31.6)	13 (68.4)		37.8 ± 31.6	
Brown or Yellow	131 (74.4)	32 (24.4)	99 (75.6)		33.3 ± 28.9	
Residence area						
Rural	30 (17.0)	8 (26.7)	22 (73.3)	1.000 ^b^	35.9 ± 28.8	0.310 ^c^
Urban	146 (83.0)	40 (27.4)	106 (72.6)		32.2 ± 31.1	
Viral load						
Below 500 copies/mL	165 (94.3)	43 (26.0)	122 (74.0)	0.145 ^b^	34.5 ± 28.9	0.208 ^c^
Above 500 copies/mL	10 (5.7)	5 (50.0)	5 (50.0)		48.1 ± 31.3	
T CD4+ Cells						
Below 350/mm^3^	34 (19.4)	11 (32.4)	23 (67.6)	0.295 ^b^	40.3 ± 29.5	0.140 ^c^
Above 350/mm^3^	141 (80.6)	37 (26.2)	104 (73.8)		34.1 ± 29.1	

Complete data on virological and immunological controls and demographic features were not obtained for two patients: one in transit and the other under follow-up in a private healthcare service. Statistical analysis: chi-square test ^a^ with Yates correction when necessary, or Fisher’s exact test ^b^; Mann–Whitney test ^c^; Kruskal–Wallis test ^d^; * significant difference.

## Data Availability

The datasets generated and/or analyzed during the current study are available from the corresponding author upon reasonable request.

## Abbreviations

The following abbreviations were used in this manuscript:

OPV	Orthopoxvirus
DNA	Deoxyribonucleic acid
VACV	Orthopoxvirus vaccinia
CPV	Orthopoxvirus cowpox
MPXV	Orthopoxvirus monkeypox
VARV	Orthopoxvirus variola
WHO	World Health Organization
BV	Bovine vaccinia
HIV	Human immunodeficiency virus
SAS	Specialized Assistance Service of Diamantina
NAbs	Neutralizing antibodies
PLHIV	People living with HIV
PRNT	Plaque reduction neutralization tests
FBS	Fetal bovine serum
MEM	Minimum Essential Medium
PFU	Plaque-forming units
BSC-40	Continuous line of African green monkey cells
BSL-3	Biosafety Level 3 Laboratory
UFMG	Universidade Federal de Minas Gerais
